# Highly Activated Neuronal Firings Monitored by Implantable Microelectrode Array in the Paraventricular Thalamus of Insomnia Rats

**DOI:** 10.3390/s23104629

**Published:** 2023-05-10

**Authors:** Jin Shan, Yilin Song, Yiding Wang, Penghui Fan, Botao Lu, Jinping Luo, Wei Xu, Luyi Jing, Fan Mo, Ruilin Hu, Yan Luo, Gang Mao, Ying Wang, Xinxia Cai

**Affiliations:** 1 State Key Laboratory of Transducer Technology, Aerospace Information Research Institute, Chinese Academy of Sciences, Beijing 100190, China; 2 School of Electronic, Electrical and Communication Engineering, University of Chinese Academy of Sciences, Beijing 100049, China; 3 Department of Anesthesiology, Ruijin Hospital, Shanghai Jiaotong University School of Medicine, Shanghai 200025, China; 4 The Fourth People’s Hospital of Jinan, Jinan 250031, China

**Keywords:** MEA, PVT, insomnia, electrophysiology

## Abstract

Insomnia is a common sleep disorder around the world, which is harmful to people’s health, daily life, and work. The paraventricular thalamus (PVT) plays an essential role in the sleep–wake transition. However, high temporal-spatial resolution microdevice technology is lacking for accurate detection and regulation of deep brain nuclei. The means for analyzing sleep–wake mechanisms and treating sleep disorders are limited. To detect the relationship between the PVT and insomnia, we designed and fabricated a special microelectrode array (MEA) to record electrophysiological signals of the PVT for insomnia and control rats. Platinum nanoparticles (PtNPs) were modified onto an MEA, which caused the impedance to decrease and improved the signal-to-noise ratio. We established the model of insomnia in rats and analyzed and compared the neural signals in detail before and after insomnia. In insomnia, the spike firing rate was increased from 5.48 ± 0.28 spike/s to 7.39 ± 0.65 spike/s, and the power of local field potential (LFP) decreased in the delta frequency band and increased in the beta frequency band. Furthermore, the synchronicity between PVT neurons declined, and burst-like firing was observed. Our study found neurons of the PVT were more activated in the insomnia state than in the control state. It also provided an effective MEA to detect the deep brain signals at the cellular level, which conformed with macroscopical LFP and insomnia symptoms. These results laid the foundation for studying PVT and the sleep–wake mechanism and were also helpful for treating sleep disorders.

## 1. Introduction

Sleep is a periodic physiological phenomenon of human beings, which is vital to health and life conditions. However, there are many people with sleep disorders, which have a massive impact on daily life and work [1]. Insomnia is a common sleep disorder, which means trouble falling or staying asleep, poor sleep quality, and decreased total sleep time [2]. Insomnia can affect not only energy and emotion but also health, work performance, and quality of life, which has become an urgent social and medical problem [3]. Currently, medication is the primary treatment for insomnia. Most medicines are helpful but have side effects. Therefore, exploring the neural and cellular mechanisms is significant in curing insomnia [4].

The paraventricular thalamus (PVT) is located in the subependymal of the third ventricle, which is one of the midline nuclei of the thalamus [5]. A recent study has shown that the PVT is important in regulating sleep and awakening [6]. There are some spinal projections of the PVT to regulate sleep–wake cycles, which are involved in the regulation of physiological and pathological function. The PVT receives the hypocretin (Hcrt) secreted by the lateral hypothalamus (LH) [7,8], serotonin secreted by the dorsal raphe nucleus (DRN) [9,10], and norepinephrine (NE) secreted by the locus coeruleus (LC) [5,11] to regulate the excitability of the PVT neurons and promote the degree of arousal. On the other hand, previous studies revealed that the fibers of the PVT project to the nucleus accumbens (NAc), and this pathway was essential to the regulation of the arousal state [6,12]. Melatonin is considered the drug to help with falling asleep and treat sleep disorders, and the PVT is the regulatory site for melatonin. Melatonin production can regulate the activity of PVT neurons by acting on melatonin receptors in the PVT through cerebrospinal fluid [13]. At the behavioral level, melatonin administration in the PVT promotes sleep occurrence, which is mediated primarily by MTR1 activation. Therefore, the PVT is a critical nucleus in the sleep–wake cycle, which needs to be detected and studied by high-resolution tools.

Currently, much research on deep brain nuclei is adopted via traditional microwire electrodes. Traditional nerve detection electrodes lacked enough temporal and spatial resolution. With the development of the micro-electro-mechanical system (MEMS), the microelectrode array (MEA) appeared and is now widely used in detecting electrophysiology and electrochemical signals of the brain [14]. As a multichannel and high-resolution detection tool, the MEA can be positioned more accurately in relation to the target and cause less trauma during implantation [15]. As metallic nanoparticles, platinum nanoparticles (PtNPs) are widely applied in medicine, electronics, and biotechnology due to their biocompatibility, antioxygenation, and surface chemistry [16]. Modifying PtNPs can effectively reduce the impedance of recording sites and improve the SNR (signal-to-noise ratio) because PtNPs effectively increase the specific area.

Based on the above background, we designed an experiment using an MEA to detect neural signals in the PVT of rat brains before and after insomnia, which attempted to explain the relationship of insomnia to the PVT nucleus from the perspective of electrophysiology. The MEA fit with the PVT nucleus was designed and fabricated by the MEMS to record the neuronal signals in the PVT of control and insomnia rats. The result showed neurons of the PVT were activated in insomnia. Through further analysis, in the insomnia state, the synchronicity between adjacent neurons decreased, and burst-like firing appeared in the same neurons. Our research explored the changes in neural signals of the PVT before and after insomnia from the perspective of electrophysiology. It provided new ideas for the study of the PVT and the treatment of sleep disorders.

## 2. Materials and Methods

### 2.1. Reagents and Apparatus

The saline was purchased from Shuanghe Corporation (Beijing, China). P-Chloro-DL-phenylalanine (PCPA), Arabic gum, and phosphate-buffered saline (PBS) were purchased from Sigma-Aldrich (Shanghai, China), and 1,1′-Dioctadecyl-3,3,3′,3′-tetramethylindocarbocyanine perchlorate (DiI) was purchased from Beyotime Institute of Biotechnology (Shanghai, China). The chloroplatinic acid (H2PtCl6) and lead acetate [(CH3COO)2Pb] were purchased from Sinopharm Chemical Reagent (Shanghai, China). The isoflurane and anesthesia machine for small animals were purchased from RWD Life Science Co., Ltd. (Shenzhen, China).

The stereotaxic frame (51600) was purchased from Stoelting (Wood Dale, IL, USA). A micropositioner (model 2662) was purchased from David KOPF instrument (Tujunga, CA, USA). A 128-channel neuroelectrophysiological recording system was purchased from Blackrock Microsystems (Salt Lake City, UT, USA). A clinical cryostat (Leica CM1950) for cutting brain slices was purchased from Leica Biosystems (Barrington, IL, USA). The microscopes were Olympus BX51 (Olympus Corporation, Tokyo, Japan) and Leica M205C (Leica Biosystems, IL, USA), which were used for observing the MEA and brain slices.

### 2.2. Animals

Four male Sprague Dawley (SD) rats (weighted 350 g) were used for experiments. These rats were purchased from Beijing Vital River Laboratory Animal Technology Co., Ltd. (Beijing, China). All rats were fed in the individual feeding cage within a 12/12 h dark–light cycle. The temperature was maintained at 25 ± 3 °C, and the humidity was maintained at 50–70%. The food and water were available ad libitum. All animal experiments were carried out with the permission of the Beijing Association on Laboratory Animal Care (Beijing, China) and approved by Institutional Animal Care and Use Committee at the Aerospace Information Research Institute, Chinese Academy of Sciences (AIRCAS, Beijing, China).

### 2.3. Design, Fabrication, and Modification of MEA

Based on the MEMS, we designed and fabricated a particular MEA to detect neural signals. The PVT area is located at a depth of 5.2 mm on the midline of the cerebral cross-section, and the shape of it is irregular. To match the depth, shape, and size of the PVT, an MEA with two tips was designed. There were eight equidistant electrophysiological recording sites and a 200 μm-long ground site on each tip (16 recording sites and 2 ground sites in total). Implanting the electrode vertically to the PVT broke the middle cerebral artery (MCA), which caused the rat to bleed to death. Therefore, we choose to tilt 30° for implantation.

The MEA consisted of three layers of material, including the base layer of Si (25 μm), the metal layer of Ti/Pt (30 nm/250 nm), and the insulating layer of SiO_2_/Si_3_N_4_ (300 nm/500 nm). Silicon-on-insulator (SOI) wafer was used as the base of MEA. Figure S1 shows the whole technological process of microelectrode preparation. First, a layer of 500 nm SiO_2_ was deposited in the wafer by thermal oxidation. Next, the Ti/Pt metal layer was deposited by photolithography, sputtering, and lift-off. Then, SiO_2_/Si_3_N_4_ insulating layer was deposited by plasma-enhanced chemical vapor deposition (PECVD). Through photolithography and deep reactive ion etching (DRIE), the conductive pads and sites were exposed, and the back oxide layer was etched. Finally, the etch of the back Si layer and the release of the MEA were accomplished by KOH wet etching. The individual released electrodes were connected to the printed circuit board (PCB) by an aluminum wire bond and then coated with protective room temperature vulcanization (RTV) silicone to complete the package.

**Figure 1:**
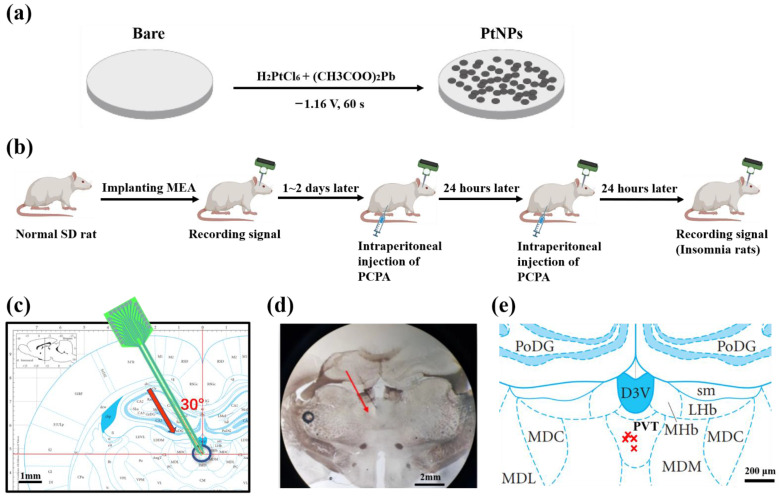
Schematic diagram of experiment process. (**a**) Scheme for electrochemical deposition of PtNPs onto microelectrode sites. (**b**) Scheme of animal experiment, including implanting MEA, insomnia modeling, and recording signals. (**c**) Implanting path of MEA to PVT at 30 degrees. The red arrow shows the direction of targeted implantation. (**d**) Histological verification of implant position. The red arrow shows the direction of experimental implantation. (**e**) The projection of implanted MEA tip. The marks of red × indicate the projections of the implanted electrode tip on the brain slices in rat brain atlas.

The electrolyte solution was prepared by mixing chloroplatinic acid (48 mM in PBS) and lead acetate (4.2 mM in PBS) in a 1:1 volume ratio, then kept for 12 h [17,18]. As shown in Figure 1a, using an electrochemical workstation (Gamry 600, Gamry Instrument, Warminster, PA, USA), the electrode tip was inserted into the electrolyte solution. PtNPs were electrodeposited on the electrode sites by chronoamperometry (−1.18 V, 60 s).

### 2.4. Surgical Operation

During the experiment, rats were anesthetized by isoflurane (3–5% for induction and 0.8–1.5% for maintenance) and fixed in the stereotaxic frame. The rat hair and scalp were cut off by a razer to expose the skull. The coordinate of craniotomy was 2.64 mm posterior and 2.8 mm lateral from the midline. Three sites of the skull were chosen to turn the skull screw as the grounding electrode and the stress point for the fixation of dental cement. To obtain firm fixation and less damage to the skull, two sites were contralateral with the skull window and one site was ipsilateral with the skull window. The positions of the sites were set to avoid the skull window. Then, the target locations of the skull were drilled by skull drill, and the scraps were cleaned by aurilave. Next, the rat and all apparatus were placed into an electromagnetic shielding box to eliminate external noise interference. The MEA was implanted at 30 degrees from the skull window (DV: 4.85 mm). Metal wires connected the shielded box (ground) and skull screws to improve the SNR. After being implanted in the target position, the MEA was fixed with dental cement. 

### 2.5. Rat Model of Insomnia

PCPA was used as a medicine to induce rat models of insomnia, which is internationally recognized [19,20]. PCPA depletes 5-HT (5-hydroxytryptamine, an important neurotransmitter that regulates the sleep–wake cycle) in the central nervous system to induce insomnia. The decrease in 5-HT caused severe insomnia symptoms. PCPA was gradually metabolized in the body, and the insomnia symptoms slowly disappeared. Therefore, signals were detected periodically after modeling the insomnia disorder.

PCPA was dissolved in PBS solution (30 mg/mL), and Arabic gum (0.1%) was added to promote dissolution. After weighing the rat, the turbid liquid of PCPA was intraperitoneally injected (1 mL/100 g of rat weight). The above operation was repeated 24 h later. On the third day, the rats developed an insomniac state. Then, spikes and LFPs were recorded as the experimental group.

### 2.6. Experimental Strategy

As shown in Figure 1b, the MEA was implanted into the PVT nucleus of the control rats. Then electrophysiological signals of the PVT neurons were recorded as the control group. After 1–2 days to recover from wounds and regain physical condition, PCPA was intraperitoneally injected twice, as described above. After the rats were in the insomnia state, electrophysiological signals of the PVT neurons were recorded again as the experimental group. Finally, the detected electrophysiological signals of the two groups were analyzed and compared to draw a reliable conclusion.

### 2.7. Signal Recording and Data Processing

Multichannel electrophysiological signals were acquired by a homemade electrophysiological recording system. The system was developed in the previous work of our group [21], which included an implantable sensor (microelectrode array), a dual-function head-stage, and a low noise detection instrument.

The MEA was connected with the homemade electrophysiological recording system. Through amplifying circuit, filter circuit, and analog to digital converter (ADC), recorded electrophysiological signals were transmitted to the upper computer. The sampling frequency was set at 30 kHz to obtain a real-time neural signal. Spikes were extracted from measured data by a high pass filter (≥250 Hz), and LFPs were extracted by a low pass filter (≤250 Hz). The special algorithm was designed for spike sorting. Cluster analysis was used for sorting according to the waveform, peak, and duration of the signals. Moreover, the single unit with a refractory period greater than 1 ms was regarded as a neuron signal. Then, different types of spikes were sorted and the noise was separated from the signals.

NeuroExplorer 4 (Nex Technologies, Hays, KS, USA) was used for further statistics and analysis of spikes and LFPs. The results were exported, and Origin (OriginLab, Northampton, MA, USA) was used to draw graphs. All data were processed as mean ± SEM. The result *p* < 0.05 (ANOVA test) was regarded as statistical significance.

### 2.8. Histological Verification of Implant Position

As the PVT was located in the deep brain region, and the oblique implantation of the MEA could easily cause errors, we used the histological section to verify that the MEA was implanted into the PVT after in vivo detection. Before implantation, the cusp of the MEA was coated with fluorochrome dye (DiI). The above step was repeated twice every half hour to ensure that DiI adhered to the MEA. After finishing the electrophysiological recording, chloral hydrate was dissolved in saline (300 mg/kg body weight) to deeply narcotize rats by intraperitoneal injection. Then, the thoracic cavity of the rat was opened with a razor and hemostatic forceps under deep anesthesia. The brain was dehydrated by injecting saline (0.9%) and paraformaldehyde (4%) into the heart artery and put in the sucrose solution (20% and 30%) in turn to fixation. Coronal sections were cut into 50 μm by a clinical cryostat. The brain slices were taken onto the glass slide. The glass slide was covered with a drop of saline in advance to spread the brain slices. Next, excess saline was removed with absorbent paper. The glass slide was observed under the stereomicroscope. After adjusting the appropriate vision to show the whole brain slices, the image of the brain slices was captured.

**Figure 2:**
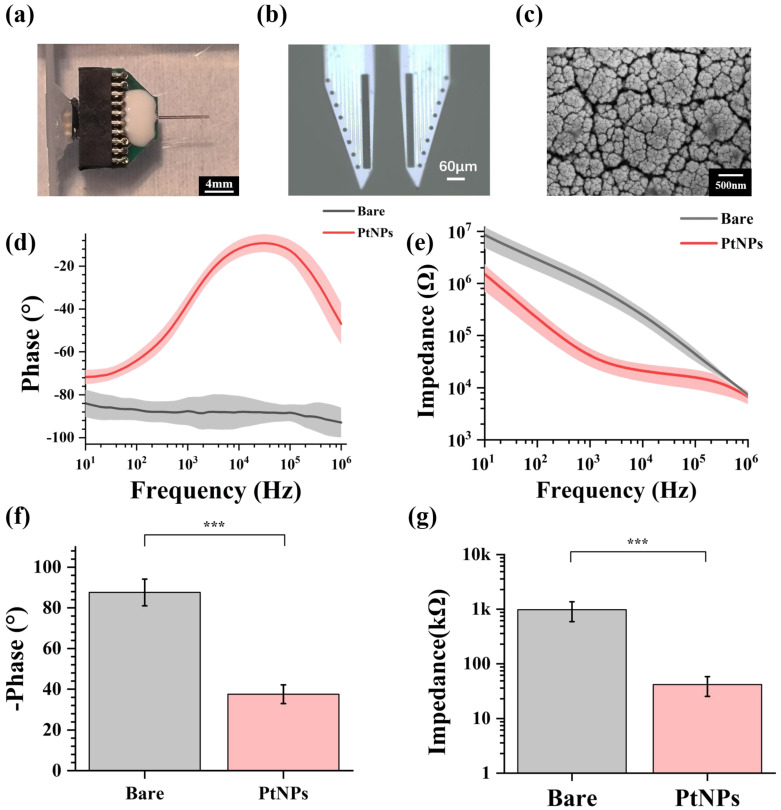
Performance characterization of PtNPs-modified MEA. (**a**) Packaged MEA with PCB. (**b**) PtNPs-modified MEA under the microscope. (**c**) The scanning electron microscopic image of PtNPs-modified electrode site at different scales. (**d**) Impedance characteristics of bare electrode sites and PtNPs-modified electrode sites from 10 Hz to 1 MHz. (**e**) Phase characteristics of bare electrode sites and PtNPs-modified electrode sites from 10 Hz to 1 MHz. (**f**) Average impedance characteristics of bare recording sites and PtNPs-modified recording sites at 1 kHz (*n* = 12, *** *p* < 0.005). (**g**) Average phase characteristics of bare recording sites and PtNPs-modified recording sites at 1 kHz (*n* = 12, *** *p* < 0.005).

The targeted position was marked on the rat brain atlas (Figure 1c), and brain slices were observed under the microscope (Figure 1d). By comparing the sections and rat brain atlas, we confirmed the rationality of the implantation method and the precision of implanting location. Figure 2e shows the projection of recording points in the atlas, which reflected the electrode implantation accuracy.

## 3. Results

### 3.1. Characterization of Electrochemically Modified Electrodes

Through design, manufacture, and modification, MEAs could be implanted for animal experiments. Figure 2a shows the fabricated and modified MEA, which was packaged with PCB, and Figure 2b shows the microscope image of PtNPs-modified MEA. All electrode sites were electroplated with black modification materials, which meant the PtNPs were modified onto the MEA sites. By observing under the scanning electron microscope (SEM) (Figure 2c), a dense layer of PtNPs was formed on the site surface, effectively increasing the specific surface area of the electrophysiological detection site. Figure S2 shows the bare and PtNPs-modified site surface under the SEM.

In this study, the electrode characterization of fabricated MEA was tested in PBS buffer (pH = 7.4) by electrochemical impedance spectroscopy (EIS). Figure 2d shows the impedance decreased significantly from 10 Hz to 1 MHz. Meanwhile, the phase angle increased, reducing the phase delay from 10 Hz to 1 MHz (Figure 2e). These results indicated that modified MEA sites displayed lower impedance and higher phase angle. By electroplating PtNPs, the impedance characteristic and phase characteristic were effectively improved.

The fundamental frequency for an action potential is 1 kHz, commonly used in neuroscience studies [22]. As shown in Figure 2f, the phase of the recording sites increased from −87.6 ± 6.6° to −37.6 ± 4.6° at 1 kHz. Figure 2g shows that the average impedance of bare electrode sites was 979.34 ± 387.77 kΩ at 1 kHz. After PtNP modification, the impedance decreased to 41.6 ± 16.4 kΩ, only 4.25% of the original impedance.

### 3.2. General Symptoms and Electrophysiological Characteristics of Insomnia Rats

After two intraperitoneal injections of PCPA, rats became weary and exhausted, and were more sensitive to light, sound, and other stimuli. Meanwhile, the rat fur became rough and dry, and their weight dropped.

**Figure 3:**
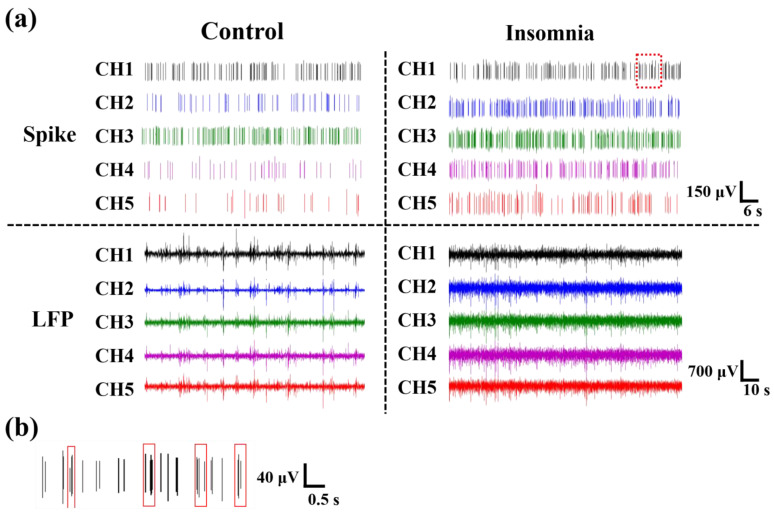
The electrophysiological signals recorded from rats. (**a**) Spikes and LFPs of different channels in control and insomnia rats. (**b**) The enlarged image of the red squares in (**a**), which was the burst-like firing in insomnia rats.

As shown in Figure 3a, we extracted original signals from the electrophysiological recording system, which included the firing at the level of a single neuron (spike) and a local cluster of neurons (LFP). Spike firing was sparser in control rats than in insomnia rats. In other words, after modeling, the spike of PVT neurons fired more densely than in control rats. Moreover, the LFP of PVT neurons fluctuated more greatly and frequently in insomnia rats than in control rats, which meant more neurons were activated in the insomnia state. Furthermore, we found there were multiple spikes (≥3) in a relatively short period of time (≤0.5 s), which is called burst-like firing (Figure 3b).

### 3.3. The Spike Firing Pattern of PVT Neurons in Control and Insomnia State 

To explore the spike characteristics in control and insomnia rats, we further analyzed the pattern of action potential discharge. Spike firing rate (number of spikes per second) was the parameter to estimate the degree of neuronal activity. The measured signal was classified as spikes and noise using principal component analysis (PCA) and k-means clustering algorithm (K-Means) [23]. According to the sorted spike, we extracted the average spike waveforms from two different states. Figure 3a showed that the average spike waveforms of the PVT were consistent, which indicated that ion channels of neurons and membrane potential were not changed after modeling the insomnia disorder. Then, the spike firing rate of the two states was calculated. As shown in Figure 3b, the firing rates of PVT neurons in control and insomnia rats were 5.48 ± 0.28 spike/s and 7.39 ± 0.65 spike/s. These findings showed that the spike firing rate of PVT neurons increased during insomnia, which meant more PVT neurons were active in insomnia than the control state. 

**Figure 4:**
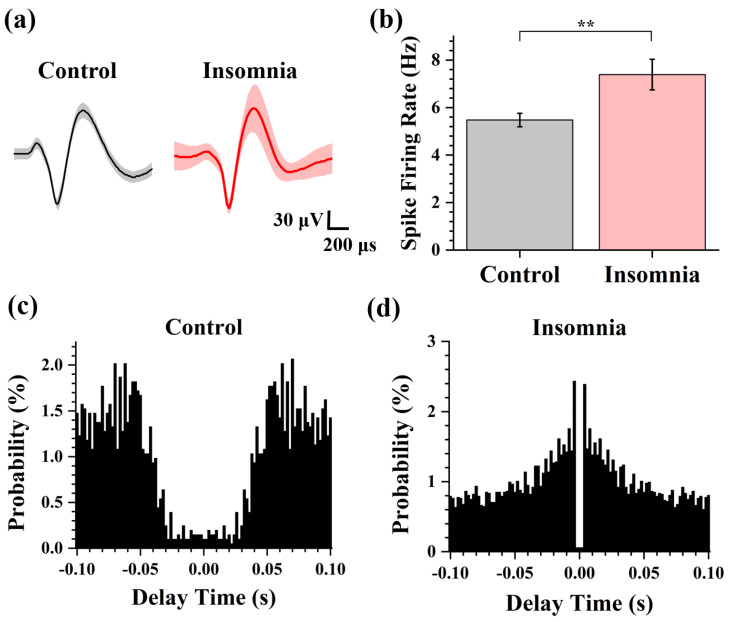
The analysis of spike in control and insomnia rats. (**a**) The average spike waveform of control and insomnia rats. (**b**) The spike firing rate in two states. (*n* = 16, ** *p* < 0.01). (**c**,**d**) The spike autocorrelograms of the typical channel in control and insomnia rats.

We also analyzed the autocorrelation of single-channel spikes and drew the autocorrelation histogram before and after model establishment. As shown in Figure 4c,d, there was a higher amplitude of the autocorrelation peak and a shorter peak latency in insomnia than in control rats, which means the delay time of spike firing in the PVT neurons was shortened, and the firing rate increased. By amplifying the measured original signal, we found that a burst or burst-like firing waveform appeared in insomnia rats, indicating a high firing rate.

### 3.4. Dynamics for the Neuronal Network of PVT in Control and Insomnia State

**Figure 5:**
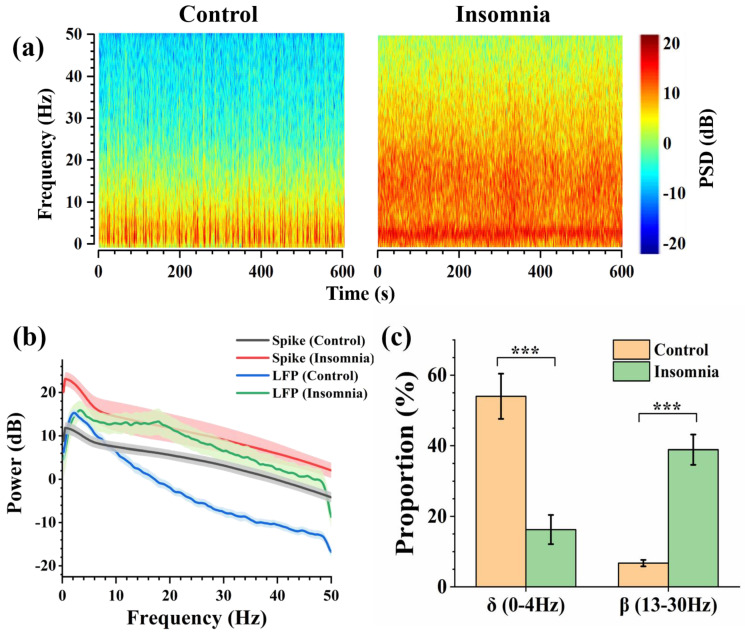
The analysis of LFP in control and insomnia rats. (**a**) Spectrogram of LFPs in typical channels for control (left) and insomnia (right) rats. (**b**) The average spike and LFP power of control and insomnia rats at 0–50 Hz. (**c**) The proportion of LFP power in the delta frequency band and the beta frequency band in two states (*n* = 10, *** *p* < 0.005).

LFP is the collection of all neurons firing in a local brain area, representing relatively macroscopic neural activity. The spectrograms (power frequency noise at 50 Hz) show the variation of frequency and PSD (power spectrum density) with time in two states. PSD reflects the energy density at a different frequency. From Figure 5a, we found an obvious line of high LFP power at 4 Hz. There was higher PSD power at the high-frequency band in insomnia rats than in control rats, and the signal energy was concentrated under 50 Hz. According to international standards, the electrophysiological frequency band was divided into the following parts: delta frequency band (0–4 Hz), theta frequency band (4–8 Hz), alpha frequency band (8–13 Hz), and beta frequency band (13–30 Hz) [24]. Figure 5b shows the LFP power of two states in 0–50 Hz. In the control state, the LFP energy was concentrated on the delta frequency band, with a peak of 15.3 dB at 2.2 Hz. The proportion of LFP power was relatively low in other frequency bands. In the insomnia state, there was also a peak of LFP power in the delta frequency band, around 15.9 dB at 3.4 Hz. Compared with the LFP power of the control state in the delta band, the frequency and amplitude of the peak in the delta frequency band both increased slightly. Moreover, the LFP power increased in the beta frequency band, with a peak of 13.3 dB at 17.8 Hz. Furthermore, the spike power was calculated based on the average spike firing rate. The spike power in the insomnia state was higher than in the normal state.

Through further analysis, the percentage of the different frequency bands in the two states was drawn as the pie charts (Figure S3). During the insomnia state, the proportion of the delta frequency band decreased from 54.02% to 16.26%. Meanwhile, the proportion of the beta frequency band increased from 6.74% to 38.88% (Figure 5c). These results showed that after modeling insomnia for rats, the LFP power of recorded LFPs increased, which means that insomnia caused an increase in PVT neuronal activity. This was also consistent with the conclusion reported in the previous study [6] that the PVT was the nucleus of promoting awakening.

### 3.5. Cross-Correlation Analysis of PVT Neurons 

**Figure 6:**
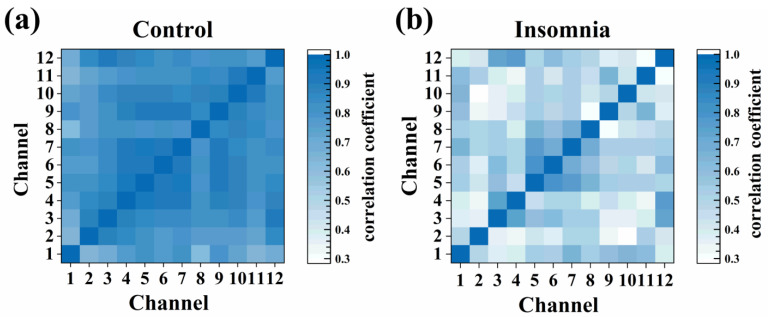
The heat map of cross-correlation coefficient for different channels in (**a**) control and (**b**) insomnia states.

In statistics, the Pearson correlation coefficient (−1 to +1) measures the correlation between two variables (linear correlation). The strength of the positive association was divided as follows: weak correlation (0.1–0.3), moderate correlation (0.3–0.5), and strong correlation (0.5–1.0). The larger the coefficient was, the stronger the correlation was. We chose and calculated the correlation coefficients of representative 12-channel signals to explore the strength of association from different channels, which are presented as a heat map (Figure 6a,b). The correlation coefficients in the insomnia state were high, and the correlation coefficients in the control state generally declined. Furthermore, the Pearson correlation coefficients were greater than 0.3 in both states, indicating specific correlations between neurons in the two states.

## 4. Discussion

Accurately acquiring electrophysiological signals from the deep brain is critical in neurosciences. As a minimally invasive tool to detect neural signals, the MEA has significant advantages for deep brain detection in vivo. The characteristics of the MEA are high precision and high temporal-spatial resolution, which help detect the signal at the cellular level (μm). The electroplated PtNPs increase the specific surface area of the conductive site and the contact with neurons, improving SNR, the performance of impedance and phase for MEA.

Recent studies have shown that the PVT is involved in sleep–wake regulation, especially in regulating and maintaining awakening. Moreover, neurons of the PVT fired more frequently during wakefulness than sleep. This finding makes us interested in the PVT and sleep–wake regulation. We chose the classical insomnia model to probe the neural signal of the PVT. The symptoms of insomnia include fatigue, an inability to concentrate, or irritability [25,26]. Compared with control rats, the average spike firing rate and spike power increased for insomnia rats. In other words, PVT neurons were activated to fire frequently in insomnia, which confirmed that the PVT was the nucleus to regulate wakefulness, and insomnia led to less sleep.

LFP sampled the activities of a relatively localized population of neurons, which meant the signal was the concentrated expression of all local neuronal discharges. We analyzed the PSD of LFP in control and insomnia rats and found that the LFP power increased at 0–50 Hz after sleeplessness. Meanwhile, the proportion of LFP power decreased in the delta frequency band and increased in the beta frequency band. Delta waves help the brain to reduce awareness of the outside environment and focus inwardly, and sleep quality is directly related to delta waves [27]. The activity of beta waves is evidently for processing information, associated with alertness, focus, and even agitation. For insomniacs, the LFP power of the delta frequency band decreased [28]. The LFP power of the beta frequency band increased and extended to the neighboring frequency bands [29]. Furthermore, there were also peaks of the average spike power in the delta frequency band, which corresponded with the peak of LFP power in the delta band. During insomnia, the spike firing rate increased, which increased spike power. It was speculated that the spike of higher power of the beta frequency in the insomnia state accumulated to the peak of LFP power in the beta frequency band. Our results also showed a lower proportion of LFP power at low frequency and a higher proportion of LFP power at high frequency in the insomnia state than in the control state. In the insomnia state, PVT neurons were highly activated at the cellular level, consistent with the macroscopical characteristics of LFP and apparent symptoms of insomnia disorder.

All physiological activities of the body were controlled and regulated by the brain, which was reflected in the neuron discharge pattern. Different physiological activities were regulated by one or more different kinds of neurons to meet body requirements. When most of the neurons were in the resting state, or they were activated in cooperation for the same physiological activity, there would be a strong synchronization between the spike firings of the neurons. The correlation coefficient was an important index to measure this synchronization. The increase in correlation coefficients meant the consistency of firing frequency, waveform, or amplitude, which indicated that the neurons maintained a similar or correlated state of physiological activities. The decline in correlation coefficients means the diversity of neuronal activity characteristics, which indicated that different kinds of neurons were activated and generated action potentials with various patterns at different times. In our research, the reduction of the cross-correlation coefficient indicated that the synchronism of discharges from different neurons in the PVT declined in the insomnia state. During sleep or anesthesia state, the body was inactive, and most neurons were at the resting stage, which induced high synchronism between neurons due to low neural activities in the brain. In the non-sleep state, more neurons were activated for more physiological activities. Therefore, there were more physiological activities of PVT neurons in the insomnia state, which meant difficulty maintaining the state of sleep.

The characteristics of insomnia were drowsiness and fatigue [30]. When the individual is at a stage of fatigue, neurons discharge by burst firing to reduce energy consumption. By current gradient stimulation to the PVT, the firing patterns of PVT neurons were observed, which included tonic firing, initial bursting, delayed firing, single spiking, and reluctant firing [31]. In our research, the observation of burst or burst-like firing is well explained.

There are two methods of signal transmission in the brain: electrical impulses firing on a single neuron (action potential) and neurotransmitters released between neurons (electrochemical signal). The neurons in the PVT are mainly excitatory glutamatergic neurons, which express vesicular glutamate transporter-2 (VGluT2). Meanwhile, there are no inhibitory GABAergic neurons in the PVT [32]. To investigate the relationship between insomnia and neurotransmitters, the changes in the concentration of glutamate in the PVT during wakefulness are worth detecting and analyzing from the electrochemical perspective. The treatment for insomnia is also an important research direction. Moreover, many nuclei associated with sleep and wakefulness were located in the deep brain region. As a regulation method for many brain diseases, DBS (deep brain stimulus) is worthy of further study regarding the DBS regulation of the sleep–wake cycle and insomnia disorder.

## 5. Conclusions

In this study, we designed and fabricated a special implantable MEA to detect the electrophysiological neural signal in the PVT and modified PtNPs on the microelectrodes to improve detection performance, which made the impedance of recording sites decrease from 979.34 ± 387.77 kΩ to 41.6 ± 16.4 kΩ at 1 kHz. Our study showed that some neurons of the PVT became activated after insomnia modeling. The electrophysiological characteristics of PVT neurons were different in control and insomnia rats. Compared with control rats, the spike firing rate increased from 5.48 ± 0.28 spike/s to 7.39 ± 0.65 spike/s, and the spike power increased. The proportion of LFP power decreased in the delta frequency band and grew in the beta frequency band. The synchronism of discharge from neighboring neurons declined in insomnia rats. These results showed that the characteristics of insomnia were consistent with macroscopic insomnia symptoms. The signals at the cellular level were detected by the fabricated MEA, which conformed with the characteristic of LFP and insomnia symptoms.

Moreover, the synchronism of different neurons was reduced. The shortened discharge interval of the same neuron and the appearance of burst-like firing indicated the generation of a fatigued state, which proved the cellular mechanism in the PVT for insomnia. Our research provided a special MEA, a tool for studying the deep brain nuclei and the nuclei associated with insomnia. Meanwhile, we explored the neuron activity of the PVT in insomnia, which provided some thoughts for further research on the sleep–wake mechanisms and the treatment of sleep disorders in the future.

## Data Availability

Data are contained within the article.

## Supplementary Materials

The following supporting information can be downloaded at: https://www.mdpi.com/article/10.3390/s23104629/s1. Figure S1: The process of MEA fabrication; Figure S2: The scanning electron image of bare and PtNPs-modified MEA sites; Figure S3: The proportion of different frequency bands in control and insomnia rats.
