# Supplementary material for: Highly Activated Neuronal Firings Monitored by Implantable Microelectrode Array in the Paraventricular Thalamus of Insomnia Rats
Source: Sensors (Basel). 2023 May 10;23(10):4629. doi: 10.3390/s23104629 (PMC10223355; doi:10.3390/s23104629)
Supplement: Supplementary file 1 [file sensors-23-04629-s001.zip › sensors-2271097-supplementary.pdf]

sensors

MDPI

Supplementary

Highly Activated Neuronal Firings Monitored by Implantable Microelectrode Array in the Paraventricular Thalamus of Insomnia Rats

Jin Shan [1,2], Yilin Song [1,2], Yiding Wang [1,2], Penghui Fan [1,2], Botao Lu [1,2], Jinping Luo [1,2], Wei Xu [1,2], Luyi Jing [1,2], Fan Mo [1,2], Ruilin Hu [1,2], Yan Luo [3], Gang Mao [4,*], Ying Wang [3,*] and Xinxia Cai [1,2,*]

1 State Key Laboratory of Transducer Technology, Aerospace Information Research Institute, Chinese Academy of Sciences, Beijing 100190, China
2 School of Electronic, Electrical and Communication Engineering, University of Chinese Academy of Sciences, Beijing 100049, China
3 Department of Anesthesiology, Ruijin Hospital, Shanghai Jiaotong University School of Medicine, Shanghai 200025, China
4 The Fourth People's Hospital of Jinan, Jinan 250031, China
* Correspondence: maogang1007@163.com (G.M.); wy10879@rjh.com.cn (Y.W.); xxcai@mail.ie.ac.cn (X.C.)

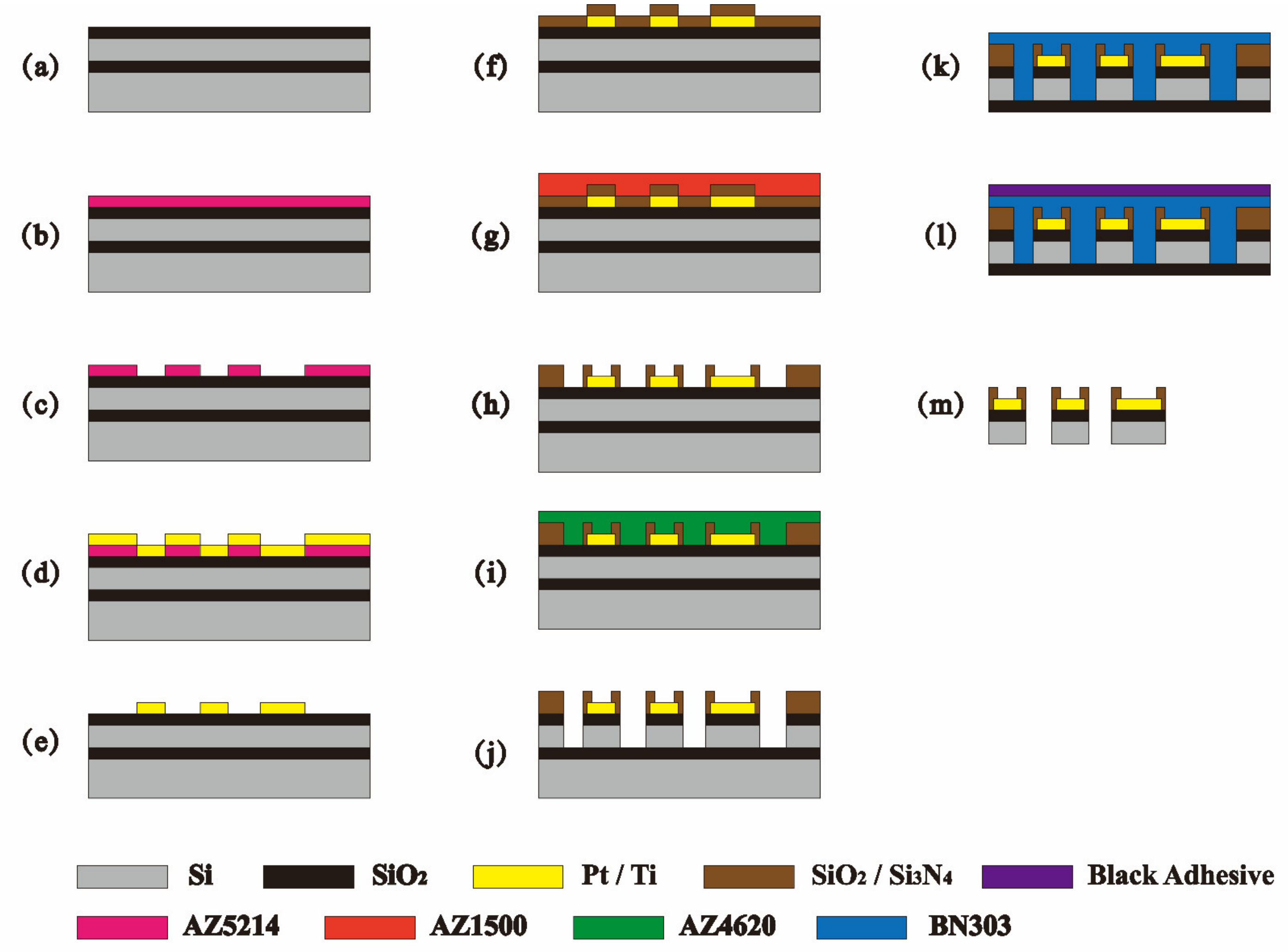

Figure S1. The process of MEA fabrication. (**a**) Deposition of SiO_2 by thermal oxidation. (**b**) Spin coating of photoresist (AZ5214). (**c**) Photoetching. (**d**) Sputtering metal layer. (Ti/Pt). (**e**) Pattern metal layer by lift-off. (**f**) Deposition of SiO_2/Si_3N_4 by PECVD. (**g**) Spin coating of photoresist (AZ1500). (**h**) Photoetching. (**i**) Spin coating of photoresist (AZ4620). (**j**) Photoetching and DRIE. (**k**) Spin coating of photoresist (BN303). (**l**) Coating of black adhesive. (**m**) Releasing electrodes by wet etching.

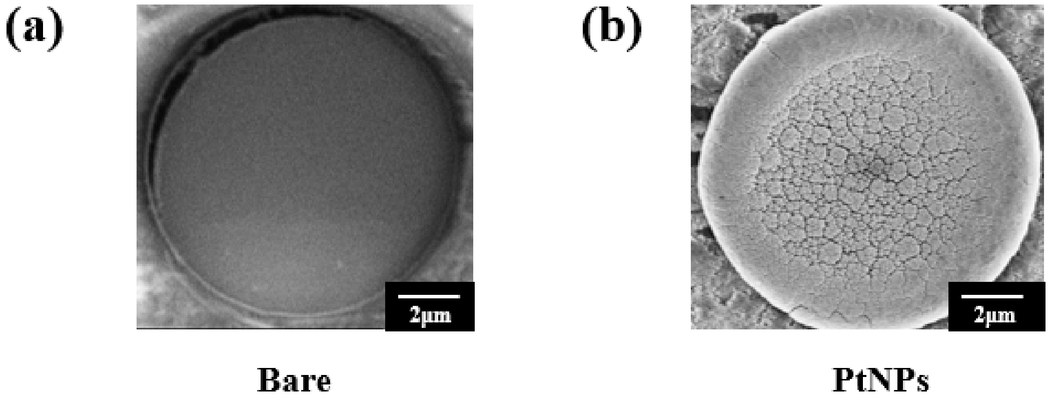

Figure S2. The scanning electron image of (**a**) bare and (**b**) PtNPs-modified MEA sites.

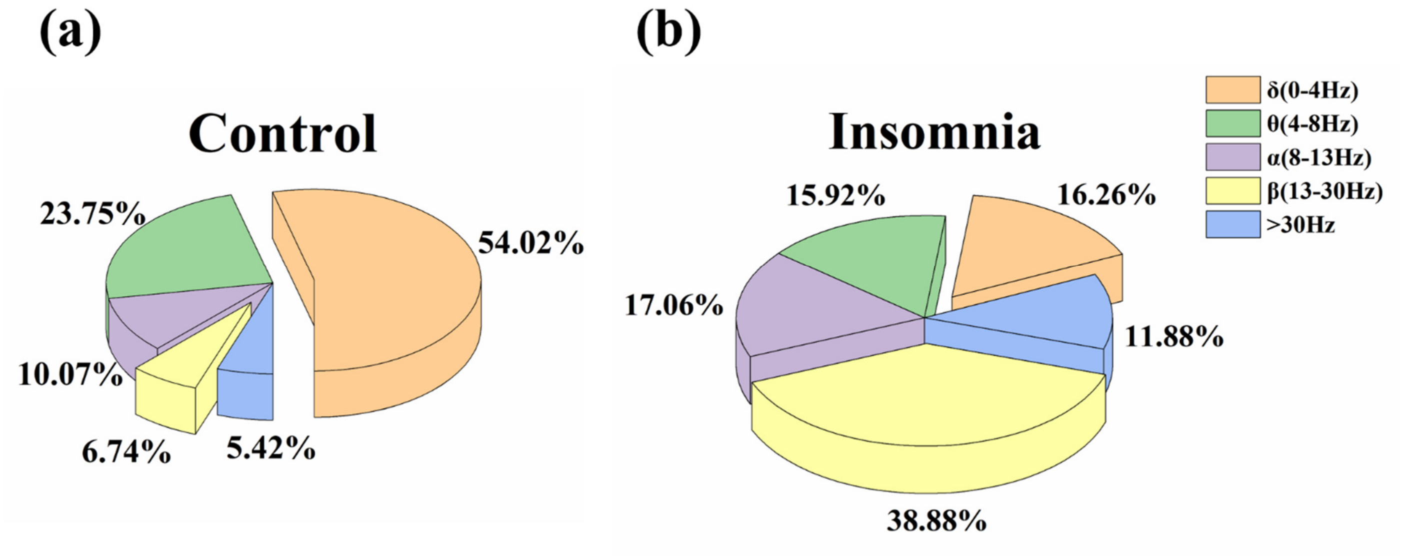

Figure S3. The proportion of different frequency bands in (**a**) control and (**b**) insomnia rats.
